# Lincosamide monotherapy treatment of methicillin-resistant *Staphylococcus aureus* pneumonia in tropical Australia: a case series

**DOI:** 10.1007/s10096-024-04816-9

**Published:** 2024-04-12

**Authors:** Stuart Campbell, Simon Smith, Josh Hanson

**Affiliations:** 1 Department of Medicine, Cairns Hospital, Cairns, Queensland Australia; 2 The Kirby Institute, University of New South Wales, Sydney, Australia

**Keywords:** Methicillin-resistant, Staphylococcus aureus, Pneumonia, Critical illness, Lincosamides, Antibiotic therapy.

## Abstract

Existing recommended first-line antibiotic agents for MRSA pneumonia have several shortcomings. We reviewed 29 cases of community- and hospital-acquired MRSA pneumonia managed at our hospital. Lincosamide monotherapy was administered to 21/29 (72%) and was the predominant antibiotic regimen (> 50% course duration) in 19/29 (66%). Patients receiving lincosamide-predominant monotherapy were no more likely to die or require intensive care unit admission than patients receiving vancomycin-predominant monotherapy (5/19 (26%) versus 4/7 (57%), *p* = 0.19); 5/7 (71%) patients admitted to ICU and 4/5 (80%) bacteraemic patients received lincosamide-predominant monotherapy. MRSA pneumonia can be safely treated with lincosamide monotherapy if the isolate is susceptible.

**Supplementary Information:**

The online version contains supplementary material available at 10.1007/s10096-024-04816-9.

## Introduction

Methicillin-resistant *Staphylococcus aureus* (MRSA) pneumonia is uncommon but has a case-fatality rate (CFR) that can approach 45% [1]. Vancomycin or linezolid are recommended as the antibiotics of choice for the treatment of MRSA pneumonia but have several limitations [2, 3]. Vancomycin has weak bactericidal activity, minimal effect on toxin production, penetrates lung tissue poorly, requires intravenous access, can cause infusion reactions, is nephrotoxic, and needs therapeutic drug monitoring [4]. Linezolid is limited by drug-drug interactions, the potential for significant adverse effects, and highly variable pharmacokinetics [5]. Another class of antibiotics, the lincosamides are widely available, well tolerated, inexpensive, and have a high bioavailability. Some guidelines suggest that lincosamide monotherapy can be used to treat MRSA pneumonia, providing the isolate is susceptible and bacteraemia has been excluded [6–8].

In Far North Queensland, in tropical Australia, almost a third of all *S. aureus* isolates are methicillin-resistant [9]. Local clinicians routinely transition to lincosamide (lincomycin or clindamycin) monotherapy for patients with MRSA bacteraemia after confirmation of lincosamide susceptibility and exclusion of endocarditis, a strategy which has been shown to be safe and effective [10]. This retrospective study aimed to define the clinical characteristics of patients with MRSA pneumonia in a region of MRSA endemicity. The study also examined the safety and efficacy of an early switch to lincosamide monotherapy ‒ once lincosamide susceptibility was confirmed ‒ in the management of patients with MRSA pneumonia.

## Methods

The hospital’s laboratory database was interrogated to identify all patients with MRSA (*S. aureus* with in vitro cefoxitin resistance) cultured from blood, sputum, or bronchoalveolar lavage samples between 1/1/2015 and 31/12/2022. Patients were included if they had a diagnosis of acute MRSA pneumonia documented by the admitting clinician, with concurrent new changes on chest imaging and MRSA isolated from blood, sputum, or bronchoalveolar lavage. Patients with infection at a concomitant primary site (e.g. infective endocarditis) were excluded, as were those providing contaminated respiratory specimens. Cases were defined as either community-acquired pneumonia (CAP) or hospital-acquired pneumonia (HAP). Demographic, clinical, radiographic, and laboratory data were collected from the patient’s electronic medical record. Antibiotic susceptibility testing was performed with the Vitek® 2 system (bioMérieux, France) using European Committee on Antimicrobial Susceptibility Testing breakpoints. Pneumonia was considered complicated if bacteraemia, lung abscess, empyema or large parapneumonic effusion were present and considered uncomplicated in their absence [8].

Antibiotic exposure was quantified in whole days, with exposure documented whatever the dosing regimen. Time to effective antibiotic therapy was defined as the time from pneumonia diagnosis until the receipt of antibiotics active against the isolated MRSA. Antibiotic duration was calculated from either the last positive MRSA culture or the first negative culture if subsequent specimens were collected. The predominant antibiotic class was defined as the class prescribed as monotherapy for the greatest proportion of the treatment course. The primary outcome was a composite of in-hospital mortality or intensive care unit (ICU) admission. Additional description of the data collected, the definitions of each variable and details of the statistical analysis are available in the supplementary methods.

## Results

There were 29 patients who met inclusion criteria (supplementary Fig. 1). Their median (interquartile range (IQR)) age was 57 (45–74) years, 18 were male. There were 24 cases of CAP and 5 cases of HAP; there were no cases of ventilator acquired pneumonia (supplementary Table 1).

MRSA was identified only in sputum or bronchoalveolar lavage in 22/29 (76%), in both respiratory samples and blood in 4/29 (14%) and blood culture only in 3/29 (10%). Resistance to trimethoprim/sulfamethoxazole was identified in 2/29 (7%) isolates, while resistance to lincosamides was detected in only 1/29 (3%). Most patients (19/29, 66%) did not have any risk factors for MRSA pneumonia. Overall, 11/29 (38%) patients died or required ICU care. Among these 11 patients, there were 5 deaths, 4 of whom were not admitted to ICU due to pre-existing defined limitations of medical intervention (supplementary Table 2). Two patients died prior to the initiation of anti-MRSA therapy; both died within 24 h of presentation.

The patients’ clinical, laboratory, and radiological findings are presented in supplementary Table 3. In multivariable analysis that included demographics and comorbidities associated with ICU admission or death, only temperature (OR (95% CI): 0.1 (0.01–0.55), *p* = 0.008) and diabetes mellitus (OR (95% CI): 15.0 (1.1-205.1), *p* = 0.043) were independently associated with ICU admission or death.

Treatment and outcome data are presented in Table 1. Of the 27 patients who received an antibiotic with activity against MRSA, the median (IQR) time to therapy was shorter in the patients who died or required ICU admission versus those that did not (2 (0–3) versus 4 (2–5) days, *p* = 0.01).

**Table 1:** Antibiotic exposures and clinical course of included patients grouped by outcome

		All (*n* = 29)	ICU-free survival (*n* = 18)	Died/required ICU (*n* = 11)	P-value	
Antibiotic exposure						
	No effective antibiotic therapy	2 (7%)	0	2 (18%)	0.14	
	Time to effective antibiotic therapy (days)	3 (2–4)	4 (2–5)	2 (0–3)	0.01	
	Lincosamide predominant therapy ^a^	19 (66%)	14 (78%)	5 (45%)	0.19	
	With bacteraemia	5	3	2		
	Vancomycin predominant therapy ^a^	7 (24%)	3 (17%)	4 (36%)	0.38	
	With bacteraemia	1	-	1		
	Any lincosamide therapy	21 (72%)	16 (89%)	5 (45%)	0.03	
	Any vancomycin therapy	19 (66%)	11 (61%)	8 (73%)	0.69	
Clinical course						
	Intercostal catheter inserted	4 (14%)	1 (6%)	3 (27%)	0.14	
	Video-assisted thoracoscopy	0	-	-	-	
	Renal replacement therapy ^b^	1 (3%)	1 (6%)	0	1.0	
	Intubation and ventilation	2 (7%)	0	2 (18%)	0.14	
	Vasopressors	3 (10%)	0	3 (27%)	0.045	
	Extracorporeal membranous oxygenation	0	-	-	-	
	Transferred	2 (7%)	1 (6%)	1 (9%)	1.0	
	DAMA	2 (7%)	1 (6%)	1 (9%)	1.0	
	Readmitted < 90 days	4 (14%)	3 (17%)	1 (9%)	1.0	

ICU – Intensive care unit, DAMA – patient discharged themselves from hospital against medical advice.

^a^ Predominant antibiotic regimen was defined as the class of antibiotic administered for the greatest number of days.

^b^ Excluding the single patient who was receiving long term haemodialysis.

Of the 27 patients who received MRSA-directed therapy, the median (IQR) treatment duration was 11 (7–16) days for uncomplicated and 36 (27–43) days for complicated pneumonia. Lincosamides were administered to 21/27 (78%) patients and were the predominant therapy in 19/27 (70%). Vancomycin was administered to 19/27 (70%) and was the predominant therapy in 7/27 (26%). Trimethoprim/sulfamethoxazole was administered to 2/27 (7%) and was the predominant antibiotic therapy in 1/27 (4%). No patient received linezolid. In the 19 patients who received lincosamide predominant therapy, 16 (84%) were transitioned to oral clindamycin, which was administered for a median (IQR) of 14 (8–27) days.

After excluding the 2 patients who received no effective anti-MRSA therapy, patients receiving lincosamide-predominant therapy were no more likely to die or require ICU care than those that did not receive lincosamide-predominant therapy (5/19 (26%) versus 4/8 (50%), *p* = 0.38). Patients receiving lincosamide-predominant therapy were no more likely to die or require ICU care than those that those receiving vancomycin-predominant therapy (5/19 (26%) versus 4/7 (57%), *p* = 0.19). However, this was not because patients receiving lincosamide-predominant therapy had milder disease: 5/6 bacteraemic patients who received anti-MRSA therapy ‒ and 5/7 patients who required ICU care ‒ received a lincosamide-predominant regimen.

There were only 2 documented adverse events attributed to antibiotics, one episode of diarrhoea (attributed to clindamycin, with negative *Clostridioides difficile* testing). The other reaction was nausea and vomiting which was unable to be attributed to a specific antibiotic.

## Discussion

In this region of tropical Australia, the case-fatality rate of MRSA pneumonia was 17%, comparable to that seen in other well-resourced health systems [11]. This is despite a third of the cohort living rurally, in some cases several hundred kilometres from the nearest ICU. Furthermore, 4/5 deaths occurred in patients whose comorbidity precluded escalation of intervention. Over a third of the cohort lacked risk factors for MRSA pneumonia, highlighting the importance of retaining a high index of suspicion in MRSA-endemic regions [11].

Patients receiving lincosamide-predominant monotherapy had similar outcomes to those receiving vancomycin-predominant monotherapy and none of the cases that died received lincosamide-predominant monotherapy. This was not explained by the prescription of lincosamides to patients with milder disease: 5/7 patients admitted to ICU and 4/5 bacteraemic patients who received therapy with activity against MRSA received lincosamide-predominant therapy. This aligns with previous research at our centre which demonstrated that lincosamide monotherapy in patients with MRSA bloodstream infections without endocarditis was associated with lower in-hospital mortality and fewer renal complications than vancomycin monotherapy [10].

This benefit of lincosamide therapy is, of course, partly explained by immortal time bias, patients must survive to have a susceptible isolate identified [12]. However, the results again demonstrate the safety, efficacy, and utility of lincosamide therapy in patients with severe MRSA infection who have a susceptible isolate.

Our study has limitations. The retrospective design precluded the collection of comprehensive data in all cases. Its modest size – its main limitation ‒ means that it is unable to answer several important questions, although the study remains one of the largest to examine the potential clinical utility of lincosamide monotherapy in MRSA pneumonia. Low (3%) rates of lincosamide resistance in our cohort enabled lincosamide use in most cases but may limit the generalisability to other settings: the rate of clindamycin resistance is ~ 13.6% Australia-wide and is even higher in other jurisdictions [13–15]. The initiation of lincosamide therapy in the hospital requires infectious diseases specialist approval; lincosamides may therefore be part of a bundle of care rather than being individually responsible for improved outcomes. Patients receiving vancomycin were defined as receiving therapy despite having possibly subtherapeutic serum levels and this may have resulting in poorer outcomes in the patients receiving vancomycin. However, subtherapeutic serum vancomycin levels are a common occurrence in clinical practice and the additional requirement for therapeutic drug monitoring is another limitation of vancomycin therapy [4].

In conclusion, lincosamide monotherapy appears to be a safe and effective alternative to vancomycin for patients with MRSA pneumonia who have a susceptible isolate. The study provides data to support the use of lincosamides in the treatment of MRSA pneumonia, although larger, prospective studies are necessary to define their optimal role.

### Electronic supplementary material

Below is the link to the electronic supplementary material.

Supplementary Material 1

### Electronic supplementary material

Below is the link to the electronic supplementary material.

Supplementary Material 2

## Data Availability

Data cannot be shared publicly because of the Queensland Public Health Act 2005. Data are available from the FNQ Human Research Ethics Committee (contact via email at FNQ_HREC@health.qld.gov.au) for researchers who meet the criteria for access to confidential data.
