# Supplementary material for: Lincosamide monotherapy treatment of methicillin-resistant Staphylococcus aureus pneumonia in tropical Australia: a case series
Source: Eur J Clin Microbiol Infect Dis. 2024 Apr 12;43(6):1247–50. doi: 10.1007/s10096-024-04816-9 (PMC11178561; doi:10.1007/s10096-024-04816-9)
Supplement: Supplementary file 1 — Supplementary Material 1 [file 10096_2024_4816_MOESM1_ESM.docx]

**Supplementary methods**

This study was carried out at the Cairns Hospital situated in the Far North Queensland region of tropical Australia. The hospital is the tertiary referral centre for approximately 290,000 people ‒ 17% of whom identify as First Nations Australians.

Community acquired pneumonia (CAP) was defined as infection onset <48 hours from presentation and hospital acquired pneumonia as infection onset ≥48 hours post admission or ≤7 days post hospital discharge. We defined contaminated respiratory specimens as those with >25 epithelial cells per x10 magnified microscopy field. A patient was defined as having a rural residence if they lived outside the Cairns metropolitan region.

The following risk factors for MRSA pneumonia were collected: previous MRSA colonisation, preceding respiratory viral infection, recent hospitalisation or antibiotic use, injection drug use, chronic haemodialysis, and the presence of bronchiectasis or severe pneumonia. MRSA colonisation was said to be present if MRSA had been previously isolated from any site within the last 12 months. Recent hospitalisation or recent antibiotic use was considered present is either were prescribed within 90 days prior. A suspected recent viral illness was defined as documented symptoms of an influenza-like illness in the preceding 28 days or detection of a respiratory virus in a respiratory tract sample in the same period. Recent injection drug use or chronic haemodialysis were identified from the EMR.

The Charlson comorbidity index (CCI) was calculated for each patient [1]. If a comorbidity was not documented in the medical record, it was presumed to be absent. Severe comorbidity was defined a CCI ≥5. Hazardous alcohol consumption was defined using Australian guidelines or if hazardous alcohol use was recorded in the medical record [2]. Any level of daily cigarette use was considered active smoking.

Laboratory data was collected at the time of diagnosis of MRSA pneumonia, aside from the C-reactive protein (CRP) which was taken as the peak value in the following 72 hours post diagnosis.

Data were entered into an electronic database (Microsoft Excel) and analysed using Stata (version 14.2). Groups were compared using the Wilcoxon rank sum test, Fisher’s exact test, or logistic regression as appropriate. Multivariable analysis was performed using backwards stepwise logistic regression; only variables that were significant in univariate analysis to a p<0.20 were included in the multivariable model.

Supplementary Results

**Supplementary Figure 1: Flowchart demonstrating inclusion and exclusion criteria in patients with MRSA pneumonia in Far North Queensland, tropical Australia 2015 – 2022.**


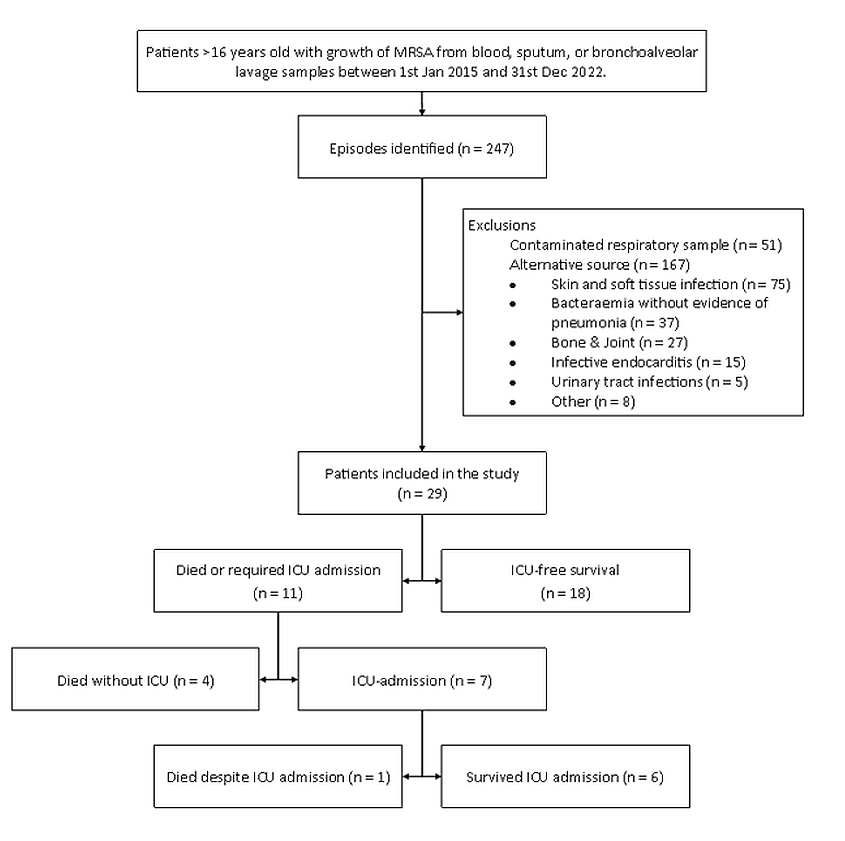


MRSA: Methicillin-resistant *Staphylococcus aureus*; ICU: Intensive care unit.

**Supplementary Table 1: Demographic factors and comorbidities of patients** **with MRSA pneumonia in Far North Queensland, tropical Australia 2015 – 2022 stratified by disease severity.**

|  |  | All | | ICU-free survival (n=18) | Died or admitted to ICU (n=11) | P-value |
| --- | --- | --- | --- | --- | --- | --- |
| Demographic | | | | | | |
|  | Median age (IQR) (years) | 57 (45-74) | 58 (48-73) | | 50 (40-78) | 0.74 |
|  | Male sex | 18 (62%) | | 12 (67%) | 6 (55%) | 0.70 |
|  | First Nations Australian | 14 (48%) | | 6 (33%) | 8 (73%) | 0.06 ^a^ |
| Admission Source | | | | | | |
|  | Care facility | 1 (3%) | | 0 | 1 (9%) | 0.38 |
|  | Rural residence | 10 (34%) | | 6 (34%) | 4 (36%) | 1.0 |
| Acquisition | | | | | | |
|  | Community acquired | 24 (83%) | | 15 (83%) | 9 (82%) | 1.0 |
|  | Hospital acquired | 5 (17%) | | 3 (17%) | 2 (18%) | 1.0 |
|  | Ventilator associated | 0 | | - | - | - |
| Risk Factors | | | | | | |
|  | Preceding viral illness | 7 (24%) | | 3 (17%) | 4 (36%) | 0.38 |
|  | Antibiotics in prior 90 days | 10 (34%) | | 4 (22%) | 6 (55%) | 0.11 ^a^ |
|  | Hospitalised in prior 90 days | 11 (38%) | | 6 (33%) | 5 (45%) | 0.70 |
|  | MRSA colonisation | 3 (10%) | | 1 (6%) | 2 (18%) | 0.54 |
| Comorbidities | | | | | | |
|  | COPD | 10 (34%) | | 6 (33%) | 4 (36%) | 1.0 |
|  | Asthma | 4 (14%) | | 3 (17%) | 1 (9%) | 1.0 |
|  | Bronchiectasis | 0 | | 0 | 0 | NA |
|  | Other chronic lung disease | 6 (21%) | | 5 (28%) | 1 (9%) | 0.4 |
|  | Obstructive sleep apnoea | 3 (10%) | | 2 (11%) | 1 (9%) | 1.0 |
|  | Any lung disease | 17 (59%) | | 11 (61%) | 5 (45%) | 1.0 |
|  | Heart failure | 5 (17%) | | 4 (22%) | 1 (9%) | 0.62 |
|  | Liver cirrhosis | 5 (17%) | | 1 (6%) | 4 (36%) | 0.05 ^a^ |
|  | Chronic kidney disease | 7 (24%) | | 4 (22%) | 3 (27%) | 1.0 |
|  | Haemodialysis | 1 (3%) | | 0 | 1 (9%) | 0.38 |
|  | Diabetes | 10 (34%) | | 4 (22%) | 6 (55%) | 0.11 ^a^ |
|  | Immunocompromised | 6 (21%) | | 3 (17%) | 3 (27%) | 0.65 |
|  | Obese (BMI > 30 kg/m^2^) | 6 (21%) | | 3 (17%) | 3 (27%) | 0.65 |
|  | Hazardous alcohol use | 5 (17%) | | 2 (11%) | 3 (27%) | 0.34 |
|  | Intravenous drug use | 1 (3%) | | 1 (6%) | 0 | 1.0 |
|  | Current smoker | 8 (28%) | | 3 (17%) | 5 (45%) | 0.20 |
|  | Median CCI (IQR) | 3 (1-7) | | 3 (1-7) | 4 (1-6) | 0.93 |
|  | No comorbidity | 5 (17%) | | 3 (17%) | 2 (18%) | 1.0 |
| ICU: Intensive Care Unit; IQR: Interquartile range, MRSA: Methicillin-resistant *Staphylococcal aureus*; COPD: Chronic obstructive pulmonary disease; BMI: Body mass index; CCI: Charlson comorbidity index.  ^a^ Signifies variables with between group differences significant to <0.20 and included in multivariate logistic analysis. | | | | | | |

**Supplementary Table 2: Characteristics of patients who died with MRSA pneumonia in Far North Queensland, tropical Australia 2015 – 2022.**

| Patient ID | | 9 | 10 | 22 | 27 | 28 |
| --- | --- | --- | --- | --- | --- | --- |
| Age (years) | | 72 | 49 | 78 | 81 | 86 |
| Sex | | Male | Female | Female | Male | Female |
| First Nations Australian | | Yes | Yes | Yes | No | No |
| RACF residence | | No | No | No | No | Yes |
| Acquisition source | | Community | Community | Hospital | Hospital | Community |
| Charlson Comorbidity Index | | 5 | 6 | 10 | 4 | 16 |
| Pneumonia severity score | | | | | | |
|  | SMART-COP | 6 | 4 | 3 | 1 | 3 |
|  | CURB-65 | 4 | 1 | 2 | 1 | 4 |
|  | CORB | 2 | 1 | 1 | 0 | 2 |
| Complicated pneumonia | | No | Yes | No | No | Yes |
| Bacteraemia | | No | Yes | No | No | Yes |
| ICU Admission | | Yes | No | No | No | No |
| Limitation on escalation | | No | Yes | Yes | Yes | Yes |
| Time to effective therapy (days) | | 0 | N/A | 3 | 3 | N/A |
| Predominate Antibiotic | | Vancomycin | Ceftriaxone | Piperacillin-Tazobactam | Piperacillin-Tazobactam | Piperacillin-Tazobactam |
| Time to death (days) | | 32 | 0 | 3 | 6 | 1 |
| RACF – Residential aged care facility; CCI - Charlson comorbidity index; ICU – Intensive care unit; N/A – not applicable (patient 10 and patient 28 did not receive any therapy with activity against MRSA, both died before the organism was identified on the day of, and the day after, presentation respectively) | | | | | | |

**Supplementary Table 3:** **Clinical findings, vital signs, laboratory values, and radiological findings of patients with MRSA pneumonia in Far North Queensland, tropical Australia 2015 – 2022 stratified by disease severity.**

|  |  | All | ICU-free survival (N=18) | Died/required ICU (N=11) | P-value |
| --- | --- | --- | --- | --- | --- |
| Clinical findings | | | | | |
|  | Symptom duration prior to diagnosis (days) | 3 (1-4) | 4 (1-5) | 2 (1-4) | 0.39 |
|  | Subjective fevers | 17 (59%) | 9 (50%) | 8 (73%) | 0.27 |
|  | Sputum production | 18 (62%) | 12 (67%) | 6 (55%) | 0.70 |
|  | Haemoptysis | 8 (28%) | 6 (33%) | 2 (18%) | 0.67 |
|  | Chest pain | 16 (55%) | 10 (56%) | 6 (55%) | 1.0 |
|  | Impaired consciousness | 5 (17%) | 2 (11%) | 3 (27%) | 0.34 |
|  | Wheeze | 3 (10%) | 3 (17%) | 0 | 0.27 |
|  | Focal signs on chest auscultation | 22 (76%) | 12 (67%) | 10 (91%) | 0.20 |
| Vital signs ^a^ | | | | | |
|  | Heart rate (bpm) | 100 (91-119) | 98 (91-120) | 107 (79-119) | 0.70 |
|  | Systolic BP (mmHg) | 122 (112-142) | 119 (110-135) | 136 (113-156) | 0.13 ^c^ |
|  | Vasopressor support | 1 (3%) | 0 | 1 (9%) | 0.38 |
|  | SaO_2_/FiO_2_ | 429 (339-452) | 435 (357-458) | 428 (323-438) | 0.17 ^c^ |
|  | Oxygen support ^b^ | 11 (38%) | 6 (33%) | 5 (45%) | 0.70 |
|  | Temperature (^o^ C) | 37.7 (36.7-38.2) | 37.9 (37.6-38.4) | 36.5 (36.4 -37.8) | 0.004 ^c^ |
| Laboratory values ^a^ | | | | | |
|  | Haemoglobin (g/L) | 126 (99-142) | 126 (109-137) | 126 (87-147) | 0.82 |
|  | White cell count (x10^9^/L) | 15.3 (10.4-23.1) | 18.2 (13.9-23.1) | 13.3 (8.2-26.3) | 0.32 |
|  | Platelet count (x10^9^/L) | 229 (175-300) | 236 (176-320) | 212 (145-246) | 0.35 |
|  | Creatinine (μmol/L) | 96 (69-152) | 81 (68-127) | 99 (73-285) | 0.32 |
|  | Bilirubin (μmol/L) | 14 (9-29) | 15 (9-30) | 14 (9-28) | 0.91 |
|  | C-reactive protein (mg/L) | 266 (145-389) | 298 (138-413) | 245 (141-316) | 0.44 |
|  | Blood glucose level (mmol/L) | 6.9 (5.7-8.4) | 7.4 (5.7-8.3) | 6.2 (4.7-22.7) | 0.32 |
|  | Positive blood culture | 8 (24%) | 3 (17%) | 5 (45%) | 0.197 ^c^ |
| Radiology | | | | | |
|  | Cavitation | 7 (24%) | 3 (17%) | 4 (36%) | 0.38 |
|  | Multilobar involvement | 21 (72%) | 12 (67%) | 9 (82%) | 0.67 |
|  | Pleural effusion present | 13 (45%) | 6 (33%) | 7 (64%) | 0.14 ^c^ |
| BP – blood pressure, SaO_2_ – Arterial oxygen saturation, FiO_2_ – Fraction of inspired oxygen.  ^a^ On day of diagnosis with the exception of CRP which was the highest value within 72h of diagnosis.  ^b^ Oxygen support signifies the requirement for supplemental oxygen at presentation.  ^c^ Signifies variables with p <0.20 and which were included in multivariate logistic analysis. | | | | | |

**References**

1. Charlson, M.E., et al., *A new method of classifying prognostic comorbidity in longitudinal studies: development and validation.* J Chronic Dis, 1987. **40**(5): p. 373-83 DOI: 10.1016/0021-9681(87)90171-8.

2. *Australian Guidelines to Reduce Health Risks from Drinking Alcohol*. 2020, Commonwealth of Australia, Canberra: National Health and Medical Research Council.
