# Supplementary figures and images for: Lincosamide monotherapy treatment of methicillin-resistant Staphylococcus aureus pneumonia in tropical Australia: a case series
Source: Eur J Clin Microbiol Infect Dis. 2024 Apr 12;43(6):1247–50. doi: 10.1007/s10096-024-04816-9 (PMC11178561; doi:10.1007/s10096-024-04816-9)

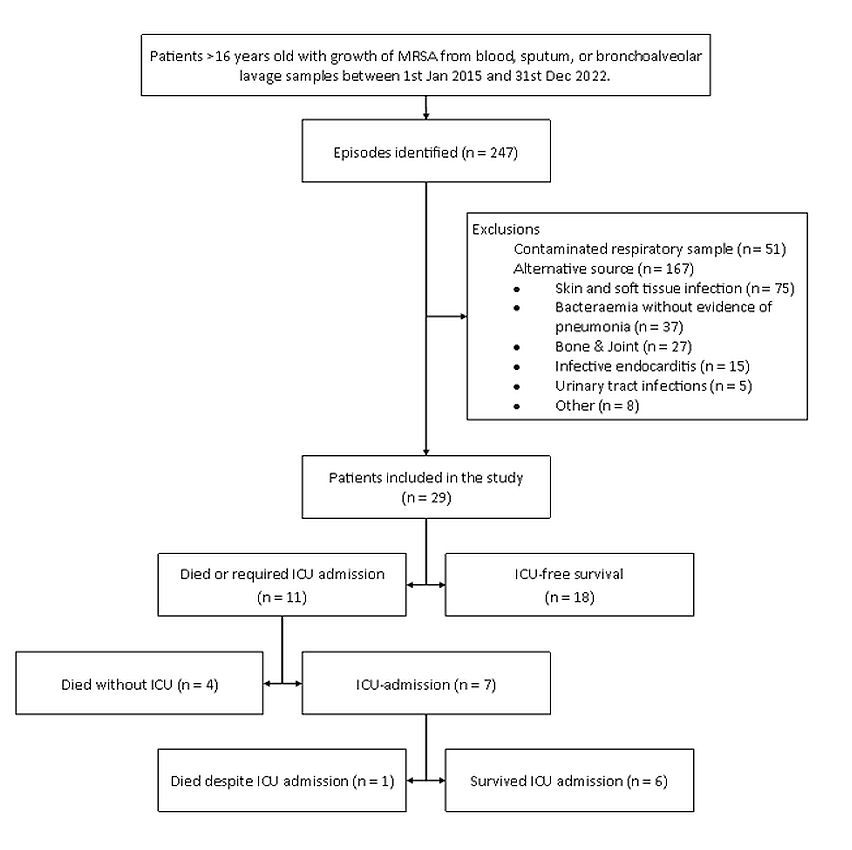

Supplement: Supplementary file 2 — Supplementary Material 2 [file 10096_2024_4816_MOESM2_ESM.tif]
